# A case report of a localized reticular erythematous mucinosis like eruption of the lower legs mimicking cutaneous larvae migrans

**DOI:** 10.1177/2050313X211003075

**Published:** 2021-03-23

**Authors:** Bret Kenny, Duane Lichtenwald, Glenda R Wright, Allison Osmond

**Affiliations:** 1 Faculty of Medicine, Memorial University, St. John’s, NL, Canada; 2 Dermatology, Saskatchewan Health Authority, Saskatoon, SK, Canada; 3 Department of Pathology and Laboratory Medicine, Saskatchewan Health Authority, Saskatoon, SK, Canada

**Keywords:** Reticular erythematous mucinosis, cutaneous mucinosis

## Abstract

Reticular erythematous mucinosis is an uncommon type of cutaneous mucinosis. Typically, reticular erythematous mucinosis affects middle-aged women and usually appears as papules and plaques on the mid-upper trunk. Histologically, biopsies of reticular erythematous mucinosis reveal increased deposition of dermal mucin and perivascular lymphocytic inflammation. Infrequently, reticular erythematous mucinosis has been reported in males and in atypical locations other than the trunk. In these instances, reticular erythematous mucinosis can present a diagnostic challenge clinically and histologically. This case describes the clinical and histologic findings of a localized variant of reticular erythematous mucinosis in a 65-year-old male patient.

## Introduction

Reticular erythematous mucinosis (REM) is classified as a primary mucinosis of the skin in which there is an increased deposition or accumulation of mucin within the dermis.^1^ REM is a rare, chronic condition that classically presents as a diffuse, erythematous, reticular rash most commonly on the chest and back (sternum, breasts, and between the scapulae). Middle-aged women are predominantly affected (2:1 female:male prevalence ratio).^2^ Despite initial observation in the 1960s,^3,4^ there is continued debate regarding the etiology of REM and whether it truly is separable from other dermatologic conditions including cutaneous manifestations of lupus erythematosus.^5^ Additional research has improved the clinical and histological understanding of REM and has helped inform practice recommendations.^6^ This case report presents the distinct case of a 65-year-old male with a *localized* variant of REM on the lower legs. This work includes a discussion of our case by situating the findings of our patient within the existing literature.

## Case presentation

A 65-year-old male presented to his primary care provider in Saskatchewan, Canada with complaints of erythematous, mildly pruritic lesions on the lower legs, ankles, and feet. The lesions appeared 3 years ago after a trip to Vietnam and the patient was concerned the lesions could be signs of a tropical disease. Based on clinical examination and recent travel history, the patient was treated for presumed cutaneous larva migrans (i.e. hookworm) and tinea corporis (i.e. body ringworm) with mebendazole as well as an overlapping course of clotrimazole and betamethasone dipropionate. Some of the lesions cleared to a satisfactory extent but relapsed and did not respond to additional courses of mebendazole. Subsequently, the patient was referred to an outpatient dermatology clinic after approximately three years since onset. The patient’s only relevant past medical history was a recent diagnosis of polymyalgia rheumatica (PMR) 6 months prior to dermatology referral, and the patient had been started on prednisone (40 mg PO once daily with appropriate taper to 5 mg PO once daily). The patient noted an appreciable improvement of the lesions while taking 40 mg/day of prednisone. However, the lesions continued to increase and decrease in severity once a maintenance dose of 5 mg/day of prednisone was achieved.

**Figure 1.**
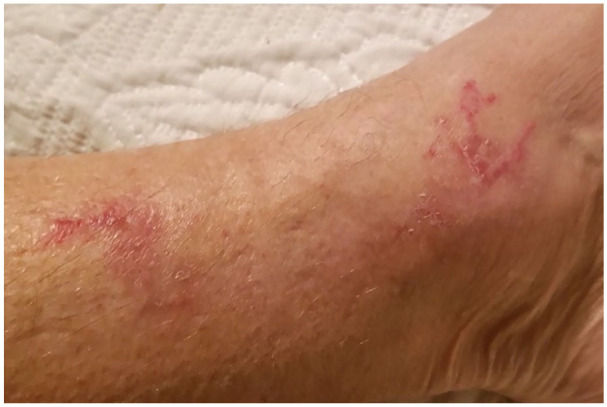
Serpiginous, erythematous plaques with slight scale located on the medial aspect of the lower left leg and ankle.

**Figure 2.**
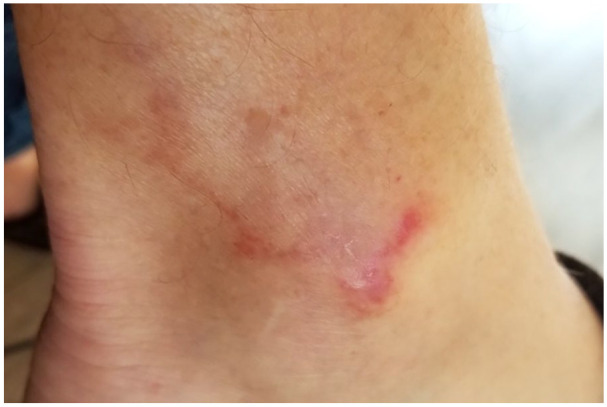
Improvement of lesions presented in Figure 1 with reduced erythema and scale.

**Figure 3.**
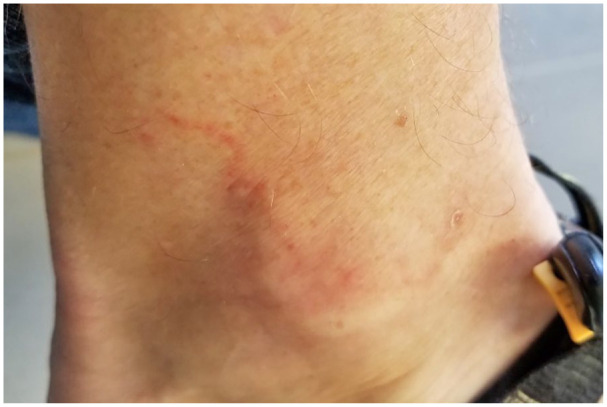
Near complete resolution of lesions presented in Figures 1 and 2 over a time period of approximately 3–4 months.

Dermatological examination indicated a changing lesion presentation over the course of approximately 3 years since initial onset. The patient provided photos at the time of consultation that revealed serpiginous, erythematous lesions approximately 3–5 cm long and 2–3 mm wide on the right lateral lower leg and ankle. These lesions cleared by the time of presentation to the dermatology clinic, and at that time skin examination revealed few subtle pink, scaly patches on the left shin and lateral ankle that were round-oval in shape with no central clearing. Follow-up 4 months later showed clearing of previous lesions and the development of a new 2–3 cm annular, dusky, erythematous plaque without scale or purpura on the right lateral ankle. Figures 1–3 present an example of representative lesions of the left medical ankle at stages from most to least severe.

The clinical differential diagnosis at the time of dermatological examination included connective tissue disease, dermal mucinosis, granuloma annulare, annular erythema centrifugum, and lichen planus mainly.

**Figure 4.**
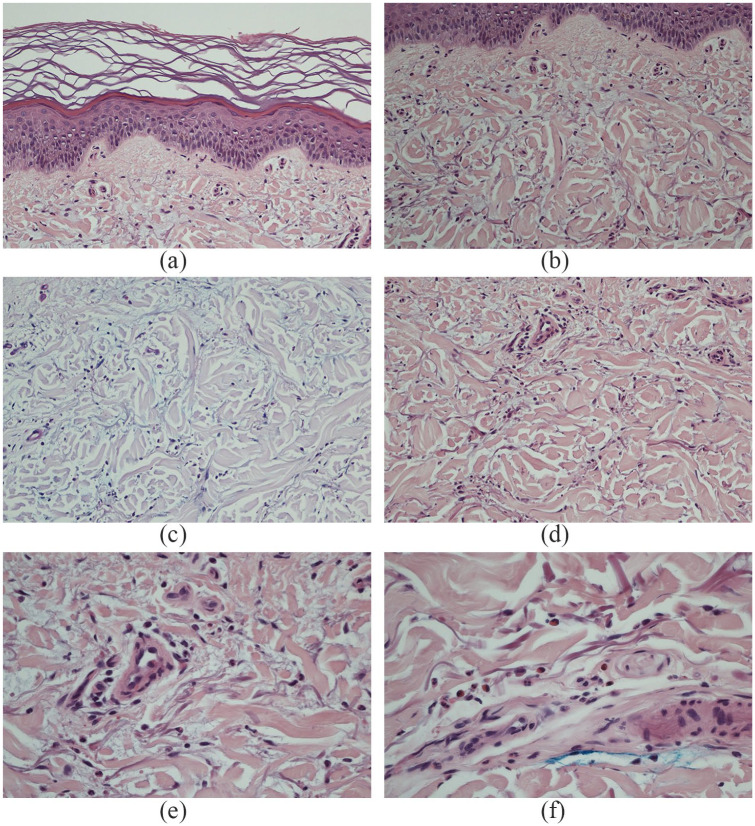
Sections shows (a) intact surface epithelium and (b and d) stringy dermal mucin with disrupted collagen fibres (e and f). (c) PAS/AB histochemical stains highlight stringy dermal mucin between collagen bundles.

Histologically, sections of the right lateral ankle punch biopsy showed stringy, Alcian blue positive superficial dermal mucin, disordered, fragmented collagen bundles (Figure 4(a)–(d)) and background scant superficial perivascular lymphohistiocytic inflammation. Small collections of eosinophils and neutrophils were appreciated on high power (40×) (Figure 4(e) and (f)). Pertinent negatives included absence of palisading histiocytes, granulomas, giant cells, necrotic dermal collagen, granulomas, vasculopathic findings, and parasites. Multiple levels through the tissue block were performed. The surface epithelium was unremarkable and intact (Figure 4(a)), free of necrotic keratinocytes, interface change, and basement membrane thickening. The deep subcutis was unremarkable. Periodic acid–Schiff (PAS) histochemical stains were also negative for fungal organisms (not shown). Histologic findings in keeping with erythema annulare centrifugum such as tight ‘coat-sleeve’ perivascular lymphocytes were absent. A diagnosis of dermal mucinosis was made, and localized REM was considered. Common histologic findings of REM include decreased amount of collagen, fragmentation of elastic fibres, increased mucin deposition in the superficial dermis, and perivascular lymphocytic infiltration within the dermis. Occasionally rare intact interstitial neutrophils are seen. Generally, the epidermis has been described as normal. These histologic findings are consistent with our case and interestingly, despite long standing treatment, these pertinent histologic features remained appreciable.

Less common histologic findings associated with REM include mildly thinned epidermis with reduced rete ridges,^3,7^ dermal vacuolated cells with a clear cytoplasm and a large basophilic hyperchromatic nucleus,^8^ dermal and dermoepidermal junction deposits of IgA, IgM, and C3,^9^ and intracytoplasmic tubular aggregates in endothelial and periendothelial cells of dermal vessels, as well as dermal histiocytes and epidermal keratinocytes via electromicroscopy.^10–13^

Laboratory investigations with normal results performed at the time of dermatologic consultation included anti-nuclear antibody (ANA), extracted nuclear antigen (ENA) screen, and complete blood count (CBC). In addition, a fungal scraping was performed with a negative result via direct microscopy and culture sample.

## Case report

This case represents a noteworthy and uncommon clinical presentation of superficial cutaneous dermal mucinosis most consistent with an atypical, localized variant of REM. The vast majority of reported REM cases present as a reticular, macular to papular, erythematous rash on the chest (sternum and/or breasts), and between the scapulae.^1,2,5^ Most commonly, the distribution is diffuse and symmetrical. Localized variants of REM, particularly of the lower extremities, are rare. Other documented, localized variants of REM include observations not only on the lower extremities but also on the upper extremities, trunk, face, and gums.^14^

A particularly interesting aspect of the clinical evolution of our case was the changing nature of the lesions which made the clinical and histologic diagnosis challenging. Typically, diffuse presentations of REM have been described to wax and wane over a long-time course, up to 30 years in some cases,^15^ during which patients would experience episodes of remission and exacerbation. The variable presentation of our patient’s lesions (i.e. the resolution and development of lesions in multiple anatomic sites) has not been described within existing literature for localized variants of REM. However, the morphology of the lesion is consistent with case reports that describe annular and/or arciform REM lesions with palpable borders.^16^ Given the continued topical treatment for presumed cutaneous larvae migrans infection and oral steroids for PMR, this may have contributed to the unusual morphologic appearance of the lesions clinically. Ultimately, the evolution and morphology of our patient’s lesions mimicked other more common dermatoses, which delayed diagnosis and prompted dermatology consultation.

The etiology of REM is still not understood; however, leading theories include a primary disorder of mucin deposition, a viral infection,^8,10–12,17^ an idiopathic photodermatosis,^18^ and an immune disorder.^19^ The increased deposition of mucin is thought to arise as a product from fibroblasts surrounding perivascular, postcapillary venules,^20^ or dermal dendrocytes.^21^ Our patient has displayed moderate improvement with a mid-potency topical steroid thought to inhibit fibroblast function. Variable response to topical steroids has been reported in many cases of REM and suggests that fibroblasts may not be involved in the pathophysiology.

The association of REM with immune disorders (systemic lupus erythematosus, hashimoto’s thyroiditis, diabetes mellitus type I), common provoking factors (UV and heat exposure, sex hormone levels),^20,22^ and neoplasia (lung, breast, colon) support the notion that antigenic stimulation and disordered immune response play a role in the pathogenesis of REM.^16^ Interestingly, our patient was diagnosed with PMR 6 months prior to dermatology consultation. A similar case has been reported by Del Pozo and colleagues of a 33-year-old female who was initially diagnosed with PMR and presented with a large atypical localization of REM on her right arm and was eventually diagnosed with systemic lupus erythematosus approximately 6 years later via the ARA criteria.^23^ The diagnosis of PMR in our case lends further credence to immune function dysregulation as a potential etiology of REM.

This interesting and uncommon case presented a diagnostic challenge. Factors including the patient’s age (65 years old), sex (male), and lesion evolution and morphology are not typically representative of classic REM. However, the patient’s past medical history of autoimmune disease (PMR), and histology are quite consistent with a localized variant of REM. Ultimately, close correlation of dermatologic and histologic findings was required to produce a diagnosis in this case and highlights the need for clinical and pathologic correlation in dermatology, especially in cases with atypical presentations.
